# Enhanced extractive text summarization framework for low-resourced Urdu language

**DOI:** 10.1371/journal.pone.0341596

**Published:** 2026-02-09

**Authors:** Shahzad Nazir, Muhammad Asif, Shahbaz Ahmad, Hanan Aljuaid, Shahbaz Ahmad

**Affiliations:** 1 Department of CS & IT, Baba Guru Nanak University, Nankana Sahib, Punjab, Pakistan; 2 Department of Computer Science, National Textile University, Faisalabad, Punjab, Pakistan; 3 Computer Sciences Department, College of Computer and Information Sciences, Princess Nourah bint Abdulrahman University (PNU), Riyadh, Saudi Arabia; 4 Faculty of Computer Science and Information Technology, The Superior University, Lahore, Pakistan; University of Lahore - Raiwind Road Campus: The University of Lahore, PAKISTAN

## Abstract

This era has witnessed an enormous increase in textual corpus available in digital form. Therefore, an intelligent mechanism is required to extract the essential information. This task is performed using an automatic text summarization that converts the text into a shorter form while the semantics are preserved. The popular languages of the world such as English, Chinese etc. have well-developed text summarization models. However, for low-resourced languages such as Urdu, well-established methods are missing. This research work proposes improved supervised and unsupervised extractive text summarization models. A large-scale dataset containing text documents and their human-annotated extractive summaries has been created. In the supervised approach, fifteen features are extracted against each sentence in the text. Further, to reduce the computational complexity, feature reduction is performed. Multiple machine learning and deep learning models are employed, including the proposed model. In an unsupervised approach, four different models are utilized. Further, the high results-producing model is incorporated with the top three features that significantly contributed to the supervised approach. The results are evaluated using ROUGE scores. Experimental results demonstrate that the proposed supervised and unsupervised approaches outperform existing Urdu text summarization methods, achieving approximately 7% and 12% improvements in ROUGE scores, respectively.

## 1 Introduction

In the current digital era, there has been an enormous increase in textual data generation. Each day, more than 2.5 quintillion bytes of data are produced, and almost 90% of all existing data has been created within the past two years [1]. During the last five years, the total amount of digital data has grown ninefold, and it is expected to reach 65 trillion gigabytes by 2030 [2]. Nearly 97.8% of this information is in text or HTML format [3]. A similar trend is visible in the Urdu language. Urdu is spoken by more than 230 million people and is the 21st most spoken first language in the world [4]. More than 1.28 million web pages are also available in Urdu [5]. Retrieving important information from such a massive digital corpus has therefore become a major challenge.

Automatic text summarization (ATS) helps reduce large volumes of text into short, meaningful summaries that preserve the main content [6]. ATS techniques are generally divided into two types: abstractive and extractive [7]. Abstractive summarization generates new sentences that express the key ideas in a coherent way. Extractive summarization, in contrast, selects the most relevant sentences from the original text based on their importance scores [8]. Well-developed extractive summarization systems exist for high-resource languages such as English, Chinese, and Spanish. However, progress in low-resource languages like Urdu remains limited. The lack of annotated datasets, linguistic tools, and evaluation benchmarks has slowed the development of accurate Urdu summarization models.

Several researchers have worked on Urdu text summarization, but the results remain unsatisfactory. Jazed et al. [9] introduced the Extractive Urdu Text Summarizer (EUTS) and achieved an F-measure of 0.594 after removing stopwords. The performance decreased when common words were removed. Nawaz et al. [10] proposed a sentence weighting method and achieved an F-measure of 0.80. Humayoun et al. [11] created a supervised dataset and applied Logistic Regression, obtaining an F-measure of 0.65. These studies show potential but the generated summaries are still not satisfactory. There is a clear need for improved techniques to produce better results for the Urdu language.

To address these issues, this study presents an enhanced extractive text summarization framework for the Urdu language. A human-annotated dataset was prepared to support experimentation. Fifteen linguistic and structural features were extracted from each sentence to capture lexical, syntactic, and positional properties. Three feature selection methods, namely Information Gain, Correlation-based Feature Selection, and Symmetrical Uncertainty, were applied to identify the most significant features. The selected features were used to train supervised hybrid models combining Convolutional Neural Networks (CNN) with Long Short-Term Memory (LSTM) and Gated Recurrent Unit (GRU) architectures. In addition, an unsupervised model was developed using the top three selected features to provide a simpler alternative. The quality of generated summaries was evaluated using the ROUGE metric [12]. The proposed supervised and unsupervised models achieved improvements of 7% and 12%, respectively, compared with previous approaches.

The key contributions of this research work are:

1. A new Urdu-language dataset was developed and manually annotated for extractive text summarization, addressing the scarcity of labeled resources for low-resourced languages.
2. A comprehensive pre-processing pipeline was designed specifically for Urdu, incorporating normalization, CRF-based tokenization, and stopword removal to handle orthographic and morphological variations effectively.
3. Two supervised hybrid models were proposed by integrating Convolutional Neural Network (CNN) layers with Long Short-Term Memory (LSTM) and Gated Recurrent Unit (GRU) architectures, enabling both local feature extraction and long-term contextual learning.
4. Fifteen linguistic, positional, and statistical features were extracted for each sentence, followed by feature selection using Information Gain, Correlation, and Symmetrical Uncertainty to enhance model efficiency and interpretability.
5. An unsupervised summarization approach was also introduced by combining the top three high-impact supervised features, reducing dependency on labeled data while maintaining competitive accuracy.
6. Experimental evaluation using ROUGE metrics demonstrated that the proposed supervised and unsupervised models achieved 7% and 12% performance improvements, respectively, over existing Urdu summarization methods.

The rest of this paper is organized as follows. Sect 2 reviews the related work on text summarization in low-resource languages. Sect 3 explains the proposed methodology, including dataset creation, feature extraction, and model training. Sect 4 presents the results and discussion, while Sect 5 concludes the study with its limitations and future directions.

## 2 Literature review

The recent decade has observed the huge generation of text corpus from numerous resources. Text summarization transforms the corpus into useful knowledge and precise information. Extractive summarization considers the sentences to have a core of the whole text and concatenate such sentences to form the summary. Massive research work has been conducted. There exist two categories of extractive text summarization 1) unsupervised and 2) supervised.

### 2.1 Unsupervised text summarization

Salton [13] is considered one of the extractive text summarization community pioneers. In 1989 TF-IDF (term frequency-inverse document frequency) based model was introduced by the author, where the document score is the ratio of the term frequency concerning the documents keeping that specific term. Here TF is the term frequency in a document, while IDF is the document’s inverse frequency. The sentences can be assigned the scores by computing TF-IDF. Goldstein et al. [14] proposed a multi-document summarization approach using the text extraction technique based on a single-document summarization approach. The authors considered four aspects for summarization 1) clustering similar passages for the identification of relevant information, 2) extracting the core concept of the source document, 3) minimizing the redundancy of words or phrases in summary, and 4) cohesion between the sentences which would be helpful for readers. The proposed model divides the document into passages and is used to index the passages. A similar approach was introduced by Vanderwende et al. [15], which is termed SUMBASIC. This probabilistic model determines the sentence significance and ultimately sorts out the summary sentences. The syntax of the Arabic language is similar to the Urdu language in the sense of writing.

For the Arabic language, Azmi et al. [16] introduced a text summarizer in 2012 that consisted of two phases. The first phase generates the summary with RST (Rhetorical Structure Theory). The second phase evaluates the summaries. This approach is similar to frequency-based techniques. This model was extended by Azmi and Altmami [17] by introducing the RST with rule-based reduction. The two native Arabic speakers evaluated the summaries. The evaluators assigned scores to summaries ranging from 1 to 5. However, the average score was observed as 4.53. An extractive text summarization approach was introduced by Qaroush et al. [18] based on semantic and statistical aspects such as 1) important phrases, 2) location of sentence and relevancy with title, 3) centrality of the sentence, 4) length of sentence, 5) cue-words, 6) positive-keywords and 7) least significant information in the document. The aspects from 1 to 4 are statistical, and the remaining are semantic-based features.

A sentence ranking algorithm for the Urdu language was introduced by Aqil et al. [19]. The authors first performed the pre-processing and counted the number of words in each sentence. Based on a count, the sentences were assigned weights and ranked according to their weights. This approach gained 64% accuracy. Aslam et al.[20] proposed Extractive Urdu Text Summarizer (EUTS). This approach also assigned weights to sentences. Initially, they performed sentence segmentation and then word segmentation. Further, the authors divided the frequency of words by word count. The evaluation of the approach was performed with a ROUGE score. The proposed approach achieved a 67 score.

### 2.2 Supervised text summarization

In recent years, transformer-based architectures [21] have significantly advanced the field of text summarization for both high- and low-resource languages [21]. Models such as BERT, T5 [22], and their multilingual counterparts (mBERT, mT5) [23] have been widely adopted due to their capability to learn contextual representations across languages. Several recent studies (2021–2025) have explored these architectures for low-resource settings. For instance, multilingual transformer frameworks have been applied to summarization tasks using mT5 and XLM-RoBERTa, achieving competitive results on non-English corpora. Similarly, BERTSUM, a fine-tuned adaptation of BERT for extractive summarization, has demonstrated strong generalization in limited-data environments.

Several deep learning-based computational predictors have been proposed across different domains to enhance feature representation and classification accuracy. Notable examples include DeepAIPs-Pred [24], TargetCLP [25], SBSM-Pro [26], DeepAIPs-SFLA [27], TargetAVP-DeepCaps [28], and PNPS-CAPSNET [29], which employ advanced architectures such as convolutional and capsule networks to capture intricate data relationships. These studies highlight the growing adoption of deep learning frameworks for predictive modeling, underscoring the potential of such architectures for improving tasks like text summarization in low-resource languages.

While transformer-based architectures now dominate text summarization research, earlier studies relied on conventional neural network approaches to address similar challenges in low-resourced languages. These models commonly utilized recurrent or convolutional layers to learn sequential dependencies without large-scale pre-training. Such approaches provided valuable insights into language-specific constraints, dataset scarcity, and morphological variations, thereby laying the groundwork for modern transformer-based solutions.

A three-phase extractive summarization approach was introduced by Jain et al. [30] using neural networks for single-document summary generation in the Punjabi language, a low-resourced language with few publicly available datasets. The Punjabi language presents several linguistic complexities, such as the use of postpositions instead of prepositions, multiple writing scripts (Shahmukhi and Gurmukhi), the absence of capitalization rules, and rich morphological diversity due to dialectal differences. The dataset, collected under the Indian Language Corpora Initiative, consisted of 30,000 Punjabi sentences annotated with Part-of-Speech tags. These sentences were then forwarded to a neural network for summary generation, where the highest-weighted sentences were selected to form the final summary. The model achieved a precision of 90.02, recall of 89.28, and an F-measure of 89.65.

Beyond Punjabi, Mohamed et al. [31] proposed a graph-based text summarization technique that integrated Explicit Semantic Analysis and Semantic Role Labeling to enhance coherence across single and multiple documents. Similarly, Muhammad Humayoun and Hwanjo Yu [32] performed preprocessing experiments that laid the foundation for Urdu extractive summarization. Their work utilized 50 Urdu-language articles and corresponding human-generated summaries while employing linguistic normalization processes such as stopword removal, lemmatization, and stemming.

More recently, the emergence of Urdu-specific pre-trained transformer models—such as *BERT-Urdu* [33], *Alif-1.0* [34], and *UrduLLaMA* [35] has substantially improved the potential for Urdu NLP tasks, including text summarization. These models are trained on large Urdu and multilingual corpora, capturing morphological and semantic nuances unique to the Urdu script. Studies leveraging these models have demonstrated notable performance gains in text classification, sentiment analysis, and abstractive summarization compared with earlier word-embedding-based approaches. Incorporating such transformer-based models into extractive or hybrid summarization frameworks represents a promising direction for advancing low-resourced language processing.

Building on these developments, Humayoun and Akhtar [11] introduced a benchmark dataset for extractive Urdu text summarization and conducted initial supervised experiments. The dataset comprises 2,649 sentences and 72,537 words, annotated for extractive summarization tasks. Using ten handcrafted features, they achieved an F1-score of 64.4%, though the results suggest further improvements are necessary to match state-of-the-art transformer-based systems.

## 3 Methodology

### 3.1 Research framework

**Fig 1:**
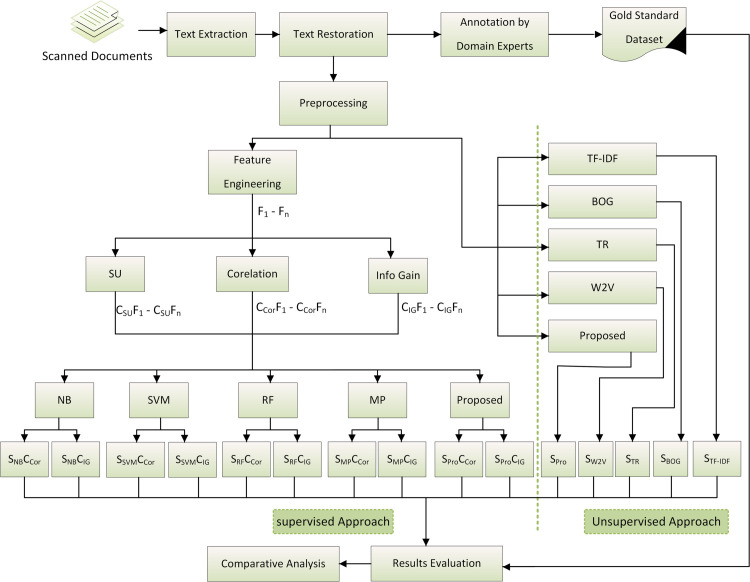
Overall proposed methodology.

This research work proposes supervised and unsupervised approaches to perform extractive text summarization for Urdu language. The overall methodology is presented in Fig 1.

### 3.2 Dataset creation

Urdu is a low-resourced language. Therefore, a large-scale dataset was created to conduct this experiment. This dataset consists of 89 different articles written in Urdu script and encompassing multiple literature aspects. These articles were in scanned form. Therefore, Google Lens was utilized for extracting text from images. However, some extracted contents were broken and incorrect. Three domain experts were hired to correct the extracted text and create extractive summaries of articles. The sentences selected by at least two annotators were included in extractive summaries. The abstractive statistics of 89 articles are presented in Table 1.

**Table 1:** Statistics of dataset.

	Number of Sentences	Number of Words	Number of Characters
Maximum	317	4765	19604
Minimum	16	450	1876
Average	88	1753	6955
Total	7753	156035	619010

As presented in Table 1, an article’s maximum number of sentences was 317. In comparison, the minimum number of sentences was 16 in an article. Likewise, other dataset statistics concerning the number of words and characters have been mentioned. The annotators performed the challenging and intense task of creating extractive summaries. It took about a quarter year to complete the dataset. The first column in the dataset represents the titles of articles. The second column contains the whole text of the articles, while the third column consists of extractive summaries. According to our knowledge, this is the largest dataset created for performing NLP tasks related to extractive summaries. We made this dataset public, so the research community may perform experiments.

### 3.3 Pre-processing techniques

Pre-processing is a crucial phase in Natural Language Processing tasks [36] as it transforms raw textual data into a consistent and analyzable format suitable for computational modeling. In this study, the pre-processing pipeline consisted of three major operations: text normalization, word tokenization, and stopword removal. The Urdu language presents several orthographic challenges because the same character can appear in different forms depending on its position in a word. Therefore, Urdu text normalization was applied to standardize these variations by converting different character shapes into a uniform representation. This step ensured consistency in character encoding and improved the accuracy of subsequent analyses.

In the tokenization stage, the continuous stream of text was segmented into tokens or words. Since Urdu is morphologically rich and lacks explicit word boundaries—spaces do not always correspond to word separations—a trained Conditional Random Field (CRF) model was utilized to perform accurate word tokenization. Finally, stopwords, which are frequent functional words that do not contribute significant semantic information [37], were removed from the corpus. Eliminating these words reduced noise and improved the effectiveness of the features extracted in later stages. Overall, this pre-processing pipeline ensured that the Urdu text was clean, standardized, and ready for efficient feature extraction and model training.

### 3.4 Supervised extractive summarization approach

Feature extraction is a critical step in the supervised extractive summarization process, as it transforms each sentence into a numerical form that reflects its linguistic and structural properties. In this study, fifteen distinct features were designed to capture multiple aspects of Urdu text characteristics. These features are grouped into five main categories: (1) sentence length–based features, which include word, character, and space ratios; (2) part-of-speech (POS)–based features, covering noun, verb, adjective, and preposition frequencies; (3) symbol-based features, accounting for punctuation and digit densities; (4) positional features, which represent the sentence order and relative position within the document; and (5) lexical features, including common word ratio, unique word count, title word ratio, and stopword ratio. Together, these features provide a comprehensive representation of each sentence, enabling the supervised model to identify those most relevant for inclusion in the extractive summary.

#### 3.4.1 Number of words, characters, and spaces.

The key elements of a sentence are the words. Longer sentences with more words are considered important and can be included in summaries. The word feature is computed using Eq (1).

$$f_{1}(s)=\frac{number\;of\;words\;in\;\text{S}}{total\;words\;in\;article}$$ (1)

The f1 feature is also termed the word ratio, which is obtained by dividing the count of sentence words by the count of all words in the article. Here ***S*** represents the sentence. Similarly, we can compute the character ratio and spaces with Eqs (2) and (3) respectively.

$$f_{2}(s)=\frac{number\;of\;characters\;in\;\text{S}}{total\;characters\;in\;article}$$ (2)

$$f_{3}(s)=\frac{number\;of\;white\;spaces\;in\;\text{S}}{white\;spaces\;in\;article}$$ (3)

A sentence may contain fewer words with a much higher count of characters. It occurs when there are longer words in a sentence.

#### 3.4.2 Count of parts-of-speech, punctuation, and numerals.

The Parts-of-speech (POS) and numerals depict the importance of a sentence. Specifically, proper nouns, verbs, adjectives, and propositions are essential. A sentence containing a high number of POS tags is considered a valuable sentence for summary creation. Likewise, we utilize more punctuation marks where important entities are described. Using Eq (4), POS and punctuation were computed

$$f_{4|5|6|7}(s)=\frac{number\;of\;(nouns|verbs|adjectives|prepositions)\;in\;\text{S}}{total\;words\;in\;\text{S}}$$ (4)

$$f_{8|9}(s)=\frac{number\;of\;(punctuations|digits)\;\;in\;\text{S}}{total\;characters\;in\;\text{S}}$$ (5)

The sentences containing digits usually present essential information in figures. The numbers can be used to mention some object’s specific year or quantity. Therefore, the sentences containing the numbers are probably part of summaries. We computed this feature with Eq (5).

#### 3.4.3 Sentence locality.

The locality of a sentence is computed with two aspects 1) the order of the sentence and 2) the position of the sentence. These features can be computed with Eqs (6) and (7).

$$f_{10}(s)=\left\{ \begin{array}{ccc} 1 & & first\_sentence\\ 2 & & second\_sentence\\ \vdots & & \vdots \end{array}\right.$$ (6)

The order of the sentence is presented by its turn. The order of the first sentence would be 1, and for the second sentence, the order will be 2. While calculating a sentence’s position, the sentence’s order is divided by the number of sentences in the whole article.

$$f_{11}(s)=\frac{order\;of\;\text{S}}{total\;sentences\;in\;article}$$ (7)

#### 3.4.4 Count of word features.

This experiment counted common words, unique words, title words, and stop words. The common words present such words of a sentence that also appeared in other sentences of the article. In comparison, unique words reflect that these words only appeared in considered sentences. These can be computed with the Eqs (8)–(10). The fourteenth feature is computing common words, while the fifteenth is computing the unique words in sentences.

$$f_{12}(s)=\frac{number\;of\;common\;words\;\;in\;\text{S}}{total\;words\;in\;\text{S}}$$ (8)

$$f_{13}(s)=\;total\;words\;in\;\text{S}\;-\;common\;words\;in\;\text{S}$$ (9)

If a sentence contains words matching the article’s title words, it can be considered significant. Similarly, the stop words can also be important. Using the equation, we can compute the title words and stop words in sentences.

$$f_{14|15}(s)=\frac{number\;of\;(title|stop)\;words\;\;in\;\text{S}}{total\;words\;in\;\text{S}}$$ (10)

The supervised dataset was created with these fifteen features which were extracted against each sentence. With such features, we also placed the selection column. In the selection column, the value 1 shows that the sentence was included in the extractive summary, and 0 depicts that the sentence was not incorporated.

Table 2 provides a comprehensive overview of the fifteen features extracted for each sentence. These features were categorized based on their linguistic and structural characteristics, serving as the input variables for the supervised extractive summarization model.

**Table 2:** Summary of extracted features for supervised extractive summarization.

ID	Feature Name	Category	Description
*f*_1_	Word Ratio	Sentence Length	Proportion of words in a sentence relative to total words in the article.
*f*_2_	Character Ratio	Sentence Length	Ratio of characters in a sentence to all characters in the article.
*f*_3_	Space Ratio	Sentence Length	Count of white spaces in a sentence relative to total white spaces in the document.
*f*_4_	Noun Frequency	POS-based	Frequency of nouns appearing in the sentence.
*f*_5_	Verb Frequency	POS-based	Frequency of verbs in a given sentence.
*f*_6_	Adjective Frequency	POS-based	Share of adjectives describing entities in the sentence.
*f*_7_	Preposition Frequency	POS-based	Number of prepositions, showing grammatical structure and complexity.
*f*_8_	Punctuation Density	Symbol-based	Count of punctuation marks emphasizing important or complex ideas.
*f*_9_	Digit Density	Symbol-based	Occurrence of numeric expressions representing factual information.
*f*_10_	Sentence Order	Positional	Sequential position of the sentence within the document.
*f*_11_	Sentence Position Ratio	Positional	Relative position of the sentence among all document sentences.
*f*_12_	Common Word Ratio	Lexical	Words shared with other sentences in the document.
*f*_13_	Unique Word Count	Lexical	Words appearing only once in the entire document.
*f*_14_	Title Word Ratio	Lexical	Words shared between the sentence and document title.
*f*_15_	Stopword Ratio	Lexical	Proportion of stopwords, indicating readability.

#### 3.4.5 Feature selection.

In supervised learning, all features may not possess the same importance [38]. Some features may significantly contribute to results, while others may not. To select the important features for the experiment, we performed feature engineering. It is a process to emphasize and eliminate potential features that produce negligible contributions to results. To select the potential features, we utilized the state-of-the-art feature engineering models 1) Symmetrical Uncertainty [39], 2) information gain [40], and 3) correlation [41]. The features obtained from the model were further utilized for sentence classification and extractive summarization.

#### 3.4.6 Sentence classification.

After selecting potential features, the sentence classification was performed. With features of each sentence, the annotation 0 describes that the sentence was not incorporated in summary while 1 conveys the sense of incorporation in extractive summary. The supervised dataset was imbalanced. Therefore, smote filter [42] was applied to create virtual instances. For class imbalance, the Synthetic Minority Over-sampling Technique (SMOTE) was applied using the imblearn library in Python. The method used k=5 nearest neighbors, a random state of 42, and an oversampling strategy of 1.0 to achieve a balanced dataset between the minority (selected sentences) and majority (non-selected) classes. This approach preserved the feature-space relationships while synthetically generating realistic minority class samples. Further, the dataset was split into a 60-40 ratio. The 60% sentences were used for training the model. In contrast, the remaining 40% sentences were selected for model testing. The classification of sentences was performed with machine learning and deep learning models. Among different state-of-the-art machine learning models, we employed 1) Naïve Bayes [43], 2) Support Vector Machine [44], 3) Random Forest [45], and 4) multilayer perceptron [46]. We also proposed models 1) CNN with LSTM and 2) CNN with GRU. These models classified the sentences and produced promising results. The features selected by Symmetrical Uncertainty, Information gain, and Correlation models were fed to machine learning and deep learning models.

### 3.5 Unsupervised extractive summarization approach

In contrast to supervised models, unsupervised models use to learn different patterns from instances. Unsupervised models are not provided with target values, and they learn concise data representations for data generation or exploration. This research work was done by utilizing multiple unsupervised models 1) TF-IDF [47], 2) Bag of Words [48], 3) TextRank [49], and 4) Word2vec [50].

After preprocessing extractive summaries were generated by utilizing these unsupervised models. Subsequently, an unsupervised model was also proposed. The top three features that significantly contributed to supervised approach were selected. These three features were incorporated into the high results producing unsupervised model. After combining the top supervised features with the unsupervised model, the extractive summaries were generated, and the results were evaluated. The code and the dataset have been uploaded on Github site (https://github.com/Shahzad-Nazir/Extractive_Urdu_Summarization).

### 3.6 Evaluation metrics

The generated extractive summaries were evaluated using ROUGE Score [12]. This score compares the generated summary by the model with the human-produced summary. ROUGE scores were considered for this experiment. In ROUGE, the Precision, recall, and f-measure are computed to evaluate the text summarization approach [51].

$$precision=\frac{number\;of\;overlapping\;words}{total\;words\;in\;generated\;summary}$$ (11)

$$recall=\frac{number\;of\;overlapping\;words}{total\;words\;in\;reference\;summary}$$ (12)

As presented in the Eqs (11)–(13), the precision is the ratio of overlapping words over the total number of words in model generated summary. Here, overlapping words describes the number of common words in model generated summary and reference summary. In contrast, the recall is computed by dividing the common number of words by the total number of words in the reference summary. The F-measure score is calculated through precision and recall.

$$F-measure=2\;\times \;\frac{\left(precision\;\times \;recall\right)}{\left(precision\;+\;recall\right)}$$ (13)

The results produced by supervised, unsupervised and the proposed approach would be compared with state-of-the-art.

## 4 Results and discussions

### 4.1 Supervised model evaluation

In the supervised approach, we first performed the pre-processing on the dataset. While executing pre-processing, normalization and word tokenization were performed. Further, 15 features were extracted from each sentence. In the Table 3, the abstract of 13 features has been mentioned. The Order-No and Sentence-Position features are derived that are based on sentence location. The words were tagged with POS. The maximum punctuation symbols in a sentence were 19, while the minimum was 0. Similarly, a sentence maximum contains 153 words and the minimum two words.

**Table 3:** Statistical analysis of extractive features of sentences.

Feature Count	Minimum Value	Maximum Value	Average Value
Punctuation	0	19	0.39
Words	2	153	20.1
Characters	3	623	79.8
Stop-words	0	100	9.81
Nouns	0	21	1.38
Verbs	0	32	4.54
Adjectives	0	30	2.43
Prepositions	0	63	6.56
Overlapping words	2	128	15.3
Unique words	0	48	4.19
Title Words	0	13	0.59
Digits	0	20	0.09
White spaces	1	152	19.3

Further, the most potential features were identified by performing feature engineering. This task was performed with 1) Symmetrical Uncertainty, 2) Information Gain, and 3) Correlation. The top 11 features were selected from each of the models. Scores are presented in Table 4.

**Table 4:** Scores of feature selecting algorithms.

Features	Information Gain	Correlation	Symmetrical Uncertainty
Punctuation	0	0.0459	0
Words	0.02251	0.159	0.0174
Characters	0.01871	0.1159	0.01486
Stop-words	0.00478	0.082	0.00532
Nouns	0.01992	0.1674	0.01533
Verbs	0	0.0457	0
Adjectives	0	0.0167	0
Prepositions	0.00203	0.0337	0.00273
Overlapping words	0	0.0436	0
Unique words	0.00251	0.056	0.00326
Title Words	0.00333	0.0643	0.0039
Digits	0.02093	0.1684	0.04423
White spaces	0.00692	0.094	0.00642
Sentence Position	0.01312	0.0199	0.02514
Sentence Order	0.02075	0.086	0.02314

Information Gain and Symmetrical Uncertainty selected the same features. Information Gain emphasizes sentence words, while Gain Ratio selects the count of numerals as the top-ranked feature. The correlation feature selection algorithm incorporated the count of punctuation and eliminated sentence position. However, nine features were same that were selected by all three feature selection models as 1) words, 2) characters, 3) stop-words, 4) nouns, 5) unique words, 6) title words, 7) numerals, 8) white spaces and 9) sentence order. The top eleven features selected by each feature selection model were further classified using machine learning and deep learning models. In machine learning, SVM, Naive Bayes, Random Forest, and Multilayer Perceptron were utilized. In comparison, we combined CNN [52] with GRU [53] and LSTM [54] to create two deep-learning models. These models classified the sentences and computed their weighted precision, recall, f-measure, and accuracy. The features selected by Information Gain and Correlation were fed into these models. The results of these selected features are presented in Table 5.

**Table 5:** ROUGE scores of supervised summaries (mean and standard deviation over multiple runs).

ROUGE Scores	Techniques	Measures	SVM	NB	LR	MP	CNN +LSTM	CNN+GRU
**ROUGE-1**	**Information Gain**	Precision	0.756 ±0.012	0.540 ±0.018	0.760 ±0.013	0.551 ±0.016	0.666 ±0.014	0.634 ±0.015
		Recall	0.525 ±0.010	0.686 ±0.012	0.519 ±0.011	0.708 ±0.017	0.627 ±0.015	0.644 ±0.013
		F-measure	0.620 ±0.011	0.605 ±0.013	0.617 ±0.012	0.620 ±0.014	0.646 ±0.011	0.639 ±0.012
	**Correlation**	Precision	0.762 ±0.014	0.522 ±0.017	0.754 ±0.013	0.810 ±0.016	0.780 ±0.012	0.664 ±0.015
		Recall	0.525 ±0.010	0.713 ±0.011	0.482 ±0.014	0.496 ±0.013	0.531 ±0.012	0.635 ±0.012
		F-measure	0.621 ±0.011	0.603 ±0.013	0.588 ±0.012	0.615 ±0.012	0.632 ±0.010	**0.650** ±0.011
**ROUGE-2**	**Information Gain**	Precision	0.573 ±0.010	0.401 ±0.012	0.589 ±0.011	0.550 ±0.013	0.408 ±0.012	0.388 ±0.010
		Recall	0.357 ±0.009	0.451 ±0.010	0.365 ±0.009	0.376 ±0.012	0.367 ±0.010	0.378 ±0.011
		F-measure	0.440 ±0.010	0.425 ±0.011	0.451 ±0.010	0.446 ±0.011	0.386 ±0.010	0.383 ±0.010
	**Correlation**	Precision	0.578 ±0.010	0.390 ±0.011	0.589 ±0.010	0.652 ±0.012	0.528 ±0.011	0.404 ±0.010
		Recall	0.356 ±0.009	0.457 ±0.009	0.337 ±0.010	0.364 ±0.011	0.345 ±0.010	0.372 ±0.010
		F-measure	0.441 ±0.010	0.421 ±0.010	0.429 ±0.010	0.468 ±0.011	0.417 ±0.010	0.388 ±0.010

All values are reported as mean ± standard deviation calculated over ten independent experimental runs.

With Information Gain attributes, it can be observed that the proposed model CNN with GRU performed better than other supervised learning models. It achieved a maximum f-measure value of 0.65. Secondly, maximum f-measure was gained by CNN with LSTM 0.64. The machine and deep learning models had similar results on both sets of selected features by Information Gain and Correlation. The CNN with GRU performed better as compared to the other models. While the quantitative results demonstrate the model’s overall effectiveness, further inspection of the output reveals areas where the proposed methods still struggle

The proposed models achieved competitive ROUGE scores, a closer inspection of the results reveals several consistent error patterns. Most false negatives occurred when the model failed to identify semantically important sentences that used paraphrased or implicit expressions, resulting in lower ROUGE-2 scores due to loss of phrase continuity. False positives were often associated with sentences containing high punctuation or numerical content, which were mistakenly assigned higher importance weights. In some cases, morphological variations and tokenization inconsistencies in Urdu also led to feature distortion, particularly for verbs and compound words. Furthermore, a positional bias was observed where sentences appearing at the beginning of the document were more likely to be selected, regardless of their true significance. These observations suggest that the model’s reliance on surface-level and structural features limits its ability to capture deeper contextual meaning. Future work will focus on addressing these issues by integrating transformer-based contextual embeddings, refining Urdu tokenization, and incorporating redundancy-aware selection mechanisms to minimize such errors.

Table 6 summarizes the optimal hyperparameters used for training the proposed supervised models. The parameters were determined experimentally by tuning batch size, learning rate, and the number of hidden units to maximize validation accuracy and F-measure performance. Early stopping and dropout regularization were employed to minimize ting and improve model generalization.

**Table 6:** Optimal training parameters used for the proposed CNN+LSTM and CNN+GRU models.

Parameter	Value
Batch size	32
Learning rate	0.001
Optimizer	Adam
Dropout rate	0.3
Number of convolution filters	128
Kernel size	3
Pooling type	Max pooling (size = 2)
LSTM/GRU units	100
Activation function	ReLU and tanh
Loss function	Binary cross-entropy
Number of epochs	50
Validation split	0.2

These parameters were selected empirically based on validation performance. Early stopping was applied to prevent overfitting during training.

Overfitting was evaluated by observing the divergence between training and validation losses across epochs. The proposed models were trained with an 80:20 data split, and the validation performance was used as an early-stopping criterion. Training automatically halted when the validation loss failed to improve for a fixed number of epochs. Dropout layers and L2 regularization were also incorporated to reduce model variance and ensure stable generalization. The use of F-measure and ROUGE metrics on the unseen test set further confirmed that the models did not exhibit overfitting behavior.

### 4.2 Unsupervised model evaluation

**Fig 2:**
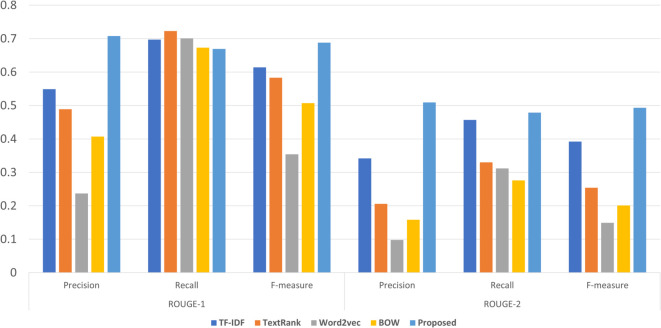
Comparing the results of different models.

This approach does not require any labeled data. The initial step of generating a summary is pre-processing. In pre-processing, we first normalized the text and performed further tokenization. The stop-words were removed from the text. Different state-of-the-art approaches were adopted, such as 1) TF-IDF, 2) TextRank, 3) BOW, and 4) Word2vec. The results achieved by these models are presented in Fig 2.

The unsupervised models produced higher ROUGE-1 scores in comparison with ROUGE-2 scores. TF-IDF model achieved a 0.61 f-measure value for ROUGE-1. Similarly, for ROUGE-2, TF-IDF results were found to be better than other models. TextRank also produced comparable results with TF-IDF. However, word2vec could not achieve acceptable results.

After analyzing supervised and unsupervised outcomes, we proposed an unsupervised model by identifying the better-performing model and the top 3 features that contribute significantly to supervised learning. This proposed model incorporated TF-IDF with the top 3 supervised features, which are 1) Numerals, 2) Nouns, and 3) word ratio.

The Fig 2, shows that the proposed unsupervised model achieved a higher F-measure than the basic TF-IDF model for ROUGE-1. On the dataset, TF-IDF gained 0.61 F-measure, while the proposed produced 0.68 F-measure value. Likewise, for ROUGE-2, the proposed model achieved slightly higher scores than other unsupervised models.

The ROUGE-1 scores are higher than the ROUGE-2 scores because ROUGE-1 measures unigram overlap between the system-generated and reference summaries, whereas ROUGE-2 evaluates bigram (two-word sequence) overlap. Since unigram matching captures broader lexical similarity and does not account for word order or local context, it generally produces higher recall and F-measure values. In contrast, ROUGE-2 is a stricter metric that reflects the model’s ability to preserve consecutive word sequences, which is more challenging in low-resource settings like Urdu, where sentence structure and word morphology exhibit greater variability.

### 4.3 Comparative analysis

**Fig 3:**
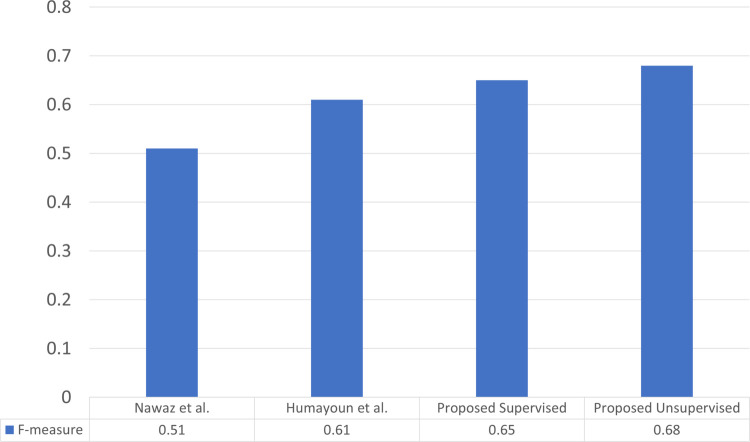
Comparison with state-of-the-art.

The results obtained from the proposed supervised and unsupervised models were compared with existing state-of-the-art Urdu summarization methods. Humayoun et al. [11] achieved an F-measure score of 0.64 using a Logistic Regression-based supervised model, while Nawaz et al. [10] proposed a sentence-weighting algorithm that attained a score of 0.80. In their approach, sentence scores were computed by dividing the number of words in each sentence by the total number of words in the corpus after stopword removal. For consistency, both the sentence-weighting algorithm and Logistic Regression were reimplemented and evaluated on the current dataset. The comparative results are illustrated in Fig 3.

As shown in Fig 3, the proposed supervised and unsupervised approaches outperform both the methods of Humayoun et al. [11] and Nawaz et al. [10]. The previous techniques did not perform consistently on the current dataset because of their limited feature representation. The sentence-weighting method relied solely on a single feature (word ratio), while the Logistic Regression model was based on a small set of manually engineered features. In contrast, the proposed supervised framework incorporated 11 linguistic, positional, and statistical features within a deep learning-based architecture, enabling the model to better capture semantic and contextual information. Similarly, the hybrid unsupervised model leveraged the top-ranked features derived from supervised evaluation, achieving higher generalization and improved summarization accuracy. These results demonstrate the effectiveness and adaptability of the proposed methods for low-resourced languages such as Urdu.

The real-life applicability of the proposed study extends to multiple Urdu-language digital ecosystems. The summarization models can be integrated into news media monitoring systems, e-court case management platforms, and social media analytics tools to automatically generate short, coherent summaries from large text corpora. Such automation can assist journalists, legal professionals, and policymakers by reducing information overload and enabling faster decision-making. Furthermore, the models can support educational and archival systems where large volumes of Urdu documents require efficient indexing and retrieval.

## 5 Limitations and future directions

Although the proposed model demonstrates encouraging performance, it has certain limitations. The approach relies on manually engineered features, which may not fully capture deeper semantic and contextual representations within Urdu text. Moreover, the dataset used for training is moderate in size, which may restrict the model’s ability to generalize across diverse domains or linguistic variations. The current framework also focuses exclusively on extractive summarization and does not incorporate abstractive or transformer-based mechanisms that could enhance coherence and semantic depth.

Future research can address these limitations by integrating transformer-based architectures such as mT5, XLM-RoBERTa, or UrduLLaMA to enable contextualized feature learning. Expanding the dataset with multi-domain and cross-lingual Urdu corpora would further improve model robustness. In addition, exploring hybrid summarization methods that combine extractive and abstractive strategies may lead to more fluent and semantically enriched summaries suitable for real-world applications.

## 6 Conclusion

The previous few years have observed enormous growth in textual data. Therefore, extracting the core information from large documents has become challenging. Automatic text summarization transforms the considerable text into short form by identifying the salient terms. This technique can be categorized into abstractive and extractive summarization. The abstractive approach learns the language and creates a summary of the text. In comparison, extractive summarization ranks the sentences by assigning scores and extracts the high-rank sentences to create the summary. Extractive summarization is considered more informative as it is based on enriched sentences. The vital languages have well-developed extractive summarization models. However, the low-resourced language, such as Urdu, has attained minor attention from researchers. The existing extractive models for the Urdu language are insignificant in producing potential summaries. This research fills the gap by proposing supervised and unsupervised extractive summarization models. A large-scale dataset was created and annotated by the domain experts. In supervised summarization, 11 features were selected after feature reduction and fed into machine learning and deep learning models. Two models were proposed: CNN with LSTM and CNN with GRU. Likewise, we considered multiple models to generate extractive summaries in unsupervised summarization. Further, an unsupervised model was proposed that incorporated the top three supervised features with high results, producing an unsupervised model. The approaches were evaluated using ROUGE scores. The supervised proposed model achieved 0.65 f-measure, and the proposed unsupervised model achieved 0.68 f-measure scores.

## Supporting information

S1 File Urdu 7 Books English Only FULL. (CSV)

## References

[1] Namjoshi J, Rawat M. Role of smart manufacturing in industry 4.0. Materials Today: Proceedings. 2022;63:475–8. 10.1016/j.matpr.2022.03.620

[2] Pani SK, Tripathy S, Butt TA, Kundu S, Jandieri G. Applications of machine learning in big-data analytics and cloud computing. River Publishers; 2022.

[3] Mehmood MA, Shafiq HM, Waheed A. Understanding regional context of world wide web using common crawl corpus. In: 2017 IEEE 13th Malaysia International Conference on Communications (MICC). 2017. p. 164–9. 10.1109/micc.2017.8311752

[4] Amjad M, Zhila A, Sidorov G, Labunets A, Butt S, Amjad HI, et al. Urduthreat@ fire2021: shared track on abusive threat identification in Urdu. In: Proceedings of the 13th Annual Meeting of the Forum for Information Retrieval Evaluation. 2021. p. 9–11.

[5] Shafiq HM, Tahir B, Mehmood MA. Towards building a Urdu language corpus using common crawl. Journal of Intelligent & Fuzzy Systems. 2020;39(2):2445–55. doi: 10.3233/jifs-179904

[6] Nenkova A, McKeown K. Automatic summarization. Foundations and Trends^®^ in Information Retrieval. 2011;5(2–3):103–233.

[7] Pilault J, Li R, Subramanian S, Pal C. On extractive and abstractive neural document summarization with transformer language models. In: Proceedings of the 2020 Conference on Empirical Methods in Natural Language Processing (EMNLP). 2020. p. 9308–19. 10.18653/v1/2020.emnlp-main.748

[8] Bhansali R, Bhave A, Bharat G, Mahajan V, Dhore ML. Abstractive text summarization of Hindi corpus using transformer encoder-decoder model. Springer Nature Singapore; 2023. p. 171–85.

[9] Muhammad A, Jazeb N, Martinez-Enriquez AM, Sikander A. EUTS: extractive Urdu text summarizer. In: 2018 Seventeenth Mexican International Conference on Artificial Intelligence (MICAI). 2018. p. 39–44. 10.1109/micai46078.2018.00014

[10] Nawaz A, Bakhtyar M, Baber J, Ullah I, Noor W, Basit A. Extractive text summarization models for Urdu language. Information Processing & Management. 2020;57(6):102383. doi: 10.1016/j.ipm.2020.102383

[11] Humayoun M, Akhtar N. CORPURES: benchmark corpus for Urdu extractive summaries and experiments using supervised learning. Intelligent Systems with Applications. 2022;16:200129. doi: 10.1016/j.iswa.2022.200129

[12] Lin CY. Rouge: a package for automatic evaluation of summaries. In: Text summarization branches out; 2004. p. 74–81.

[13] Salton G. Automatic text processing: the transformation, analysis, and retrieval of. Reading: Addison-Wesley; 1989.

[14] Goldstein J, Mittal V, Carbonell J, Kantrowitz M. Multi-document summarization by sentence extraction. In: NAACL-ANLP 2000 Workshop on Automatic Summarization. 2000.

[15] Svore K, Vanderwende L, Burges C. Enhancing single-document summarization by combining RankNet and third-party sources. In: Proceedings of the 2007 joint conference on empirical methods in natural language processing and computational natural language learning (EMNLP-CoNLL). 2007. p. 448–57.

[16] Azmi AM, Al-Thanyyan S. A text summarizer for Arabic. Computer Speech & Language. 2012;26(4):260–73. doi: 10.1016/j.csl.2012.01.002

[17] Azmi AM, Altmami NI. An abstractive Arabic text summarizer with user controlled granularity. Information Processing & Management. 2018;54(6):903–21. doi: 10.1016/j.ipm.2018.06.002

[18] Qaroush A, Abu Farha I, Ghanem W, Washaha M, Maali E. An efficient single document Arabic text summarization using a combination of statistical and semantic features. Journal of King Saud University - Computer and Information Sciences. 2021;33(6):677–92. doi: 10.1016/j.jksuci.2019.03.010

[19] Burney A, Sami B, Mahmood N, Abbas Z, Rizwan K. Urdu text summarizer using sentence weight algorithm for word processors. International Journal of Computer Applications. 2012;46(19):38–43.

[20] Muhammad A, Jazeb N, Martinez-Enriquez AM, Sikander A. EUTS: extractive Urdu text summarizer. In: 2018 Seventeenth Mexican International Conference on Artificial Intelligence (MICAI). 2018. p. 39–44. 10.1109/micai46078.2018.00014

[21] Masih S, Hassan M, Fahad LG, Hassan B. Transformer-based abstractive summarization of legal texts in low-resource languages. Electronics. 2025;14(12):2320.

[22] Enevoldsen K, Chung I, Kerboua I, Kardos M, Mathur A, Stap D. Mmteb: Massive multilingual text embedding benchmark. arXiv preprint 2025. https://arxiv.org/abs/250213595

[23] Munaf M, Afzal H, Mahmood K, Iltaf N. Low resource summarization using pre-trained language models. ACM Trans Asian Low-Resour Lang Inf Process. 2024;23(10):1–19. doi: 10.1145/3675780

[24] Akbar S, Ullah M, Raza A, Zou Q, Alghamdi W. DeepAIPs-pred: predicting anti-inflammatory peptides using local evolutionary transformation images and structural embedding-based optimal descriptors with self-normalized BiTCNs. J Chem Inf Model. 2024;64(24):9609–25. doi: 10.1021/acs.jcim.4c01758 39625463

[25] Ullah M, Akbar S, Raza A, Khan KA, Zou Q. TargetCLP: clathrin proteins prediction combining transformed and evolutionary scale modeling-based multi-view features via weighted feature integration approach. Brief Bioinform. 2024;26(1):bbaf026. doi: 10.1093/bib/bbaf026 39844339 PMC11753890

[26] Wang Y, Zhai Y, Ding Y, Zou Q. SBSM-Pro: support bio-sequence machine for proteins. Sci China Inf Sci. 2024;67(11). doi: 10.1007/s11432-024-4171-9

[27] Akbar S, Raza A, Alghamdi W, Saeed A, Ali H, Zou Q. DeepAIPs-SFLA: deep convolutional model for prediction of anti-inflammatory peptides using binary pattern decomposition of novel multiview descriptors with an SFLA approach. ACS Omega. 2025;10(32):35747–62. doi: 10.1021/acsomega.5c02422 40852276 PMC12368731

[28] Akbar S, Raza A, Zou Q, Alghamdi W, Kang X, Ali H, et al. Accelerating prediction of antiviral peptides using genetic algorithm-based weighted multiperspective descriptors with self-normalized deep networks. J Chem Inf Model. 2025;65(18):9815–30. doi: 10.1021/acs.jcim.5c01777 40901705

[29] Akbar S, Raza A, Awan HH, Zou Q, Alghamdi W, Saeed A. pNPs-CapsNet: predicting neuropeptides using protein language models and fasttext encoding-based weighted multi-view feature integration with deep capsule neural network. ACS Omega. 2025;10(12):12403–16. doi: 10.1021/acsomega.4c11449 40191328 PMC11966582

[30] Jain A, Arora A, Yadav D, Morato J, Kaur A. Text summarization technique for punjabi language using neural networks. IAJIT. 2021. doi: 10.34028/iajit/18/6/8

[31] Mohamed M, Oussalah M. SRL-ESA-TextSum: a text summarization approach based on semantic role labeling and explicit semantic analysis. Information Processing & Management. 2019;56(4):1356–72. doi: 10.1016/j.ipm.2019.04.003

[32] Humayoun M, Yu H. Analyzing pre-processing settings for Urdu single-document extractive summarization. In: Proceedings of the tenth international conference on language resources and evaluation (LREC’16). 2016. p. 3686–93.

[33] Ul Hasan M, Raza A, Rafi M. UrduBERT: a bidirectional transformer for Urdu language understanding. ACM Trans Asian Low-Resour Lang Inf Process. 2022;21:1–22.

[34] Shafique MA, Mehreen K, Arham M, Amjad M, Butt S, Farooq H. Alif: advancing Urdu large language models via multilingual synthetic data distillation. arXiv preprint 2025. https://arxiv.org/abs/251009051

[35] Fiaz L, Tahir MH, Shams S, Hussain S. UrduLLaMA 1.0: dataset curation, preprocessing, and evaluation in low-resource settings. arXiv preprint 2025. https://arxiv.org/abs/250216961

[36] Zhou M, Duan N, Liu S, Shum H-Y. Progress in neural NLP: modeling, learning, and reasoning. Engineering. 2020;6(3):275–90. doi: 10.1016/j.eng.2019.12.014

[37] Khan N, Bakht MP, Khan MJ, Samad A, Sahar G. Spotting Urdu stop words by Zipf’s statistical approach. In: 2019 13th International Conference on Mathematics, Actuarial Science, Computer Science and Statistics (MACS). 2019. p. 1–5. 10.1109/macs48846.2019.9024817

[38] Li J, Cheng K, Wang S, Morstatter F, Trevino RP, Tang J. Feature selection: a data perspective. ACM Comput Surv. 2017;50(6):1–45.

[39] Wang Q, Jiang H, Ren J, Liu H, Wang X, Zhang B. An intrusion detection algorithm based on joint symmetric uncertainty and hyperparameter optimized fusion neural network. Expert Systems with Applications. 2024;244:123014. doi: 10.1016/j.eswa.2023.123014

[40] Kurita T. Principal component analysis (PCA). Computer Vision: A Reference Guide. 2019. p. 1–4.

[41] Roobaert D, Karakoulas G, Chawla NV. Information gain, correlation and support vector machines. Feature extraction: foundations and applications. Springer. 2006. p. 463–70.

[42] Pradipta GA, Wardoyo R, Musdholifah A, Sanjaya INH, Ismail M. SMOTE for handling imbalanced data problem: a review. In: 2021 Sixth International Conference on Informatics and Computing (ICIC). 2021. p. 1–8. 10.1109/icic54025.2021.9632912

[43] Webb GI, Keogh E, Miikkulainen R. Naïve Bayes. Encyclopedia of machine learning. 2010. p. 713–4.

[44] Suthaharan S, Suthaharan S. Support vector machine. Machine learning models and algorithms for big data classification: thinking with examples for effective learning. 2016. p. 207–35.

[45] Rigatti SJ. Random forest. J Insur Med. 2017;47(1):31–9. doi: 10.17849/insm-47-01-31-39.1 28836909

[46] Ramchoun H, Ghanou Y, Ettaouil M, Janati Idrissi MA. Multilayer perceptron: architecture optimization and training. 2016.

[47] Christian H, Agus MP, Suhartono D. Single document automatic text summarization using Term Frequency-Inverse Document Frequency (TF-IDF). ComTech. 2016;7(4):285. doi: 10.21512/comtech.v7i4.3746

[48] Zhang Y, Jin R, Zhou Z-H. Understanding bag-of-words model: a statistical framework. Int J Mach Learn & Cyber. 2010;1(1–4):43–52. doi: 10.1007/s13042-010-0001-0

[49] Mihalcea R, Tarau P. Textrank: bringing order into text. In: Proceedings of the 2004 conference on empirical methods in natural language processing. 2004. p. 404–11.

[50] Church KW. Word2Vec. Natural Language Engineering. 2017;23(1):155–62.

[51] Powers DM. Evaluation: from precision, recall and F-measure to ROC, informedness, markedness and correlation. arXiv preprint 2020. https://arxiv.org/abs/2010.16061

[52] Gu J, Wang Z, Kuen J, Ma L, Shahroudy A, Shuai B, et al. Recent advances in convolutional neural networks. Pattern Recognition. 2018;77:354–77. doi: 10.1016/j.patcog.2017.10.013

[53] Dey R, Salem FM. Gate-variants of Gated Recurrent Unit (GRU) neural networks. In: 2017 IEEE 60th International Midwest Symposium on Circuits and Systems (MWSCAS), 2017. p. 1597–600. 10.1109/mwscas.2017.8053243

[54] Graves A, Graves A. Long short-term memory. In: Supervised sequence labelling with recurrent neural networks. 2012. p. 37–45.

